# Antibacterial Activities of Phenolic Compounds in *Miang* Extract: Growth Inhibition and Change in Protein Expression of Extensively Drug-Resistant *Klebsiella pneumoniae*

**DOI:** 10.3390/antibiotics13060536

**Published:** 2024-06-09

**Authors:** Pannita Anek, Sutita Kumpangcum, Sittiruk Roytrakul, Chartchai Khanongnuch, Chalermpong Saenjum, Kulwadee Phannachet

**Affiliations:** 1 Department of Microbiology, Faculty of Medicine, Chiang Mai University, Chiang Mai 50200, Thailand; pannita_a@cmu.ac.th (P.A.); sutita_kum@cmu.ac.th (S.K.); 2 National Center for Genetic Engineering and Biotechnology, National Science and Technology Development Agency, Khlong Luang, Pathum Thani 12120, Thailand; sittiruk@biotec.or.th; 3 Research Center for Innovation in Analytical Science and Technology for Biodiversity-Based Economic and Society (I-ANALY-S-T_B.BES-CMU), Chiang Mai University, Chiang Mai 50200, Thailand; chartchai.k@cmu.ac.th; 4 Research Center for Multidisciplinary Approaches to Miang, Chiang Mai University, Chiang Mai 50200, Thailand; 5 Department of Pharmaceutical Sciences, Faculty of Pharmacy, Chiang Mai University, Chiang Mai 50200, Thailand

**Keywords:** *Miang* extract, proteomic analysis, extensively drug-resistant bacteria, antibacterial activity, pyrogallol

## Abstract

The rising incidence of extensively drug-resistant (XDR) *Klebsiella pneumoniae*, including carbapenem- and colistin-resistant strains, leads to the limitation of available effective antibiotics. *Miang*, known as chewing tea, is produced from *Camellia sinensis* var. *assamica* or Assam tea leaves fermentation. Previous studies revealed that the extract of *Miang* contains various phenolic and flavonoid compounds with numerous biological activities including antibacterial activity. However, the antibacterial activity of *Miang* against XDR bacteria especially colistin-resistant strains had not been investigated. In this study, the compositions of phenolic and flavonoid compounds in fresh, steamed, and fermented Assam tea leaves were examined by HPLC, and their antibacterial activities were evaluated by the determination of the MIC and MBC. Pyrogallol was detected only in the extract from *Miang* and showed the highest activities with an MIC of 0.25 mg/mL and an MBC of 0.25–0.5 mg/mL against methicillin-susceptible *Staphylococcus aureus*, methicillin-resistant *S. aureus*, *Escherichia coli* ATCC 25922, colistin-resistant *E. coli*, and colistin-resistant *K. pneumoniae*. The effects on morphology and proteomic changes in *K. pneumoniae* NH54 treated with *Miang* extract were characterized by SEM and label-free quantitative shotgun proteomics analysis. The results revealed that *Miang* extract caused the decrease in bacterial cell wall integrity and cell lysis. The up- and downregulated expression with approximately a 2 to >5-fold change in proteins involved in peptidoglycan synthesis and outer membrane, carbohydrate, and amino acid metabolism were identified. These findings suggested that *Miang* containing pyrogallol and other secondary metabolites from fermentation has potential as an alternative candidate with an antibacterial agent or natural active pharmaceutical ingredient against XDR bacteria including colistin-resistant bacteria.

## 1. Introduction

*K. pneumoniae*, a member of the Enterobacteriaceae family, is a major causative agent associated with various community- and nosocomial-acquired infections. However, the overuse and inappropriate use of antibiotics are considered as motivating forces for bacteria to develop their antibiotic resistance abilities. One of the effective strategies is the exchange of drug resistance genes among bacteria leading to the rapid spreading of multidrug-resistant strains. The widespread extended spectrum beta-lactamase (ESBL)-producing *Klebsiella pneumoniae* together with the persistent increase in carbapenem-resistant *K. pneumoniae* cause the limitation of available antibiotic treatment options. The rising incidence of multidrug-resistant bacterial strains in the last few decades caused a crisis in global public heath for lacking effective antibiotics. In particular, carbapenem-resistant *K. pneumoniae* is categorized as one of the first priority (critical) pathogens for which new antibiotics are urgently needed by the World Health Organization (WHO) [1]. Colistin, one of the last-resort drugs, is used for the treatment of carbapenem-resistant bacterial infections despite its adverse side effects such as neurotoxicity and nephrotoxicity. The situation of insufficient effective antibiotics is worsening with the emergence of colistin-resistant bacteria including *K. pneumoniae*. Therefore, seeking compounds with activities against multidrug-resistant bacteria including carbapenem- and colistin-resistant strains is the crucial aim to solve this problem.

*Miang* also known as chewing or eating tea is commonly available in northern Thailand and made from *Camellia sinensis* var. *assamica* or Assam tea leaves using the traditional fermentation process [2]. The fermentation can be conducted either with or without the requirement of fungi growth classified as a filamentous fungi-based process (FFP) and non-filamentous fungi-based process (NFP), respectively [3]. In the fermentation process, macronutrients such as carbohydrates, lipids, and proteins in steamed tea leaves are biotransformed by microorganism metabolic activities resulting mainly in catechins and derivatives, gallic acids, and tannins [4]. Previous studies revealed that the overall phenolic compounds were increased when compared with unfermented tea leaves and showed the activities’ correlation with the inhibition of intracellular reactive oxygen species (ROS) and nitric oxide, a proinflammatory mediator, as well as radical scavenging activity. Among these compounds, pyrogallol, gallic acid, and ellagic acids were proposed to serve as the pharmacophores responsible for the bioactivities of *Miang* from the FFP and NFP [5]. However, many studies provide evidence of the antimicrobial activities of tea phenolic and flavonoid compounds, specifically catechins, but the detail of the modes of action has been inconclusive.

In this study, the compositions of phenolic and flavonoid compounds in fresh, streamed, and fermented Assam tea leaves were analyzed as well as the evaluations of the antibacterial activities of each type of tested sample extracts against multidrug-resistant and extensively drug-resistant bacterial strains. Additionally, the effects of *Miang* on the bacterial cell morphology were determined by scanning electron microscopy (SEM). Finally, the bacterial proteomic changes in response to *Miang* treatment were explored.

## 2. Results

### 2.1. Polyphenol and Flavonoid Compounds in Miang Extracts

Catechin, catechin derivatives, and related compounds including gallic acid, gallocatechin, epigallocatechin, caffeine, epicatechin, epigallocatechin gallate, gallocatechin gallate, and epicatechin gallate were detected with different concentrations in fresh, streamed, and fermented Assam tea leaves or *Miang* extracts, as exhibited in Table 1. The HPLC chromatogram of the mixed standard including catechin, catechin derivatives, and related compounds comparable to *Miang* extract is shown in Figure S1. The quantities of catechin and caffeine in all extracts were ranked the highest. However, the amount of catechin in *Miang* (17.75 ± 0.13 mg/g extract) was significantly decreased compared to fresh (36.78 ± 0.18 mg/g extract) and steamed (22.26 ± 0.17 mg/g extract) Assam tea leaf extracts. This finding contrasted with the amount of gallic acid that was higher in fermented Assam tea leaves or *Miang*. Interestingly, pyrogallol was undetectable in both fresh and streamed Assam tea leaf extracts but was found with a concentration of 4.37 ± 0.06 (mg/g extract) in *Miang*.

### 2.2. Antibiotic Resistance Profile of Clinical Isolate Bacteria

Antibiotic resistance profiles were determined by disc diffusion tests for 26 antimicrobial agents and by a broth microdilution test for colistin susceptibility (Table S1). The control strains, *Escherichia coli* ATCC 25922 and methicillin-susceptible *Staphylococcus aureus* MSSA01, were confirmed to be susceptible to all tested antibiotic agents. Gram-negative clinical isolates, *E. coli* CRE10 and *K. pneumoniae* NH54, were susceptible to less than three antibacterial categories and characterized as extensively drug resistant (XDR) bacteria. Moreover, *E. coli* CRE10 and *K. pneumoniae* NH54 were resistant to carbapenems, monobactam, and fluoroquinolone as well as colistin. *E. coli* CRE10 and *K. pneumoniae* NH54 were resistant to colistin which is one of the last-resort antibiotics, with an MIC of 4 and 16, respectively. The antibiotic resistance profile of *S. aureus* MRSA08, a methicillin-resistant bacterium, showed that it was a multidrug-resistant (MDR) strain and non-susceptible to seven out of nine antibiotics including cefoxitin, gentamicin, moxifloxacin, and trimethoprim/sulfamethoxazole.

### 2.3. Antibacterial Activity of Catechin, Catechin Derivatives, and Related Compounds, and Tested Extracts against XDR and MDR Bacteria

Crude extracts of fresh, streamed, and fermented Assam tea leaves were tested for antibacterial activities against Gram-negative XDR bacteria and methicillin-resistant *S. aureus* and evaluated according to the MIC and MBC. Among the three types of extracts, the fermented Assam tea leaves or *Miang* exhibited the highest activity of bacterial growth inhibition against all tested bacteria according to the lowest range of the MIC (≤0.5–2 mg/mL) and MBC (0.5–2 mg/mL), while the fresh and steamed Assam tea leaf extracts showed less efficiency with an MIC of 6–32 mg/mL and MBC of 8–64 mg/mL and an MIC of 1–16 mg/mL and MBC of 1–64 mg/mL, respectively, as shown in Table 2. The only exception was found in the case of the fermented and steamed Assam tea leaf extracts which exhibited the same activity against MSSA. The antibacterial activities of the three types of extracts of interest against either antibiotic-susceptible strains (*S. aureus* MSSA01 and *E. coli* ATCC 25922) or -resistant strains (*S. aureus* MRSA08 and *E. coli* CRE10) appeared to have no preference activities against either bacterial group. The antibacterial activities of various phenolic and flavonoid compounds found in *Miang* extracts including pyrogallol were determined individually. Interestingly, among the tested polyphenol and flavonoid compounds, pyrogallol showed the best antibacterial activity with an MIC of 0.1–0.25 mg/mL and MBC of 0.25–0.5 mg/mL, while other compounds showed an MIC and MBC of >2 mg/mL (Table 3). The information obtained from the analysis of bioactive compounds found in fresh, steamed, and fermented Assam tea leaves revealed that pyrogallol was detected only in fermented Assam tea leaves or *Miang*. This finding corresponded to the highest antibacterial activity of fermented Assam tea leaf extracts compared with fresh and steamed Assam tea leaf extracts.

### 2.4. The Effect of Miang Extract on Bacterial Morphology

**Figure 1:**
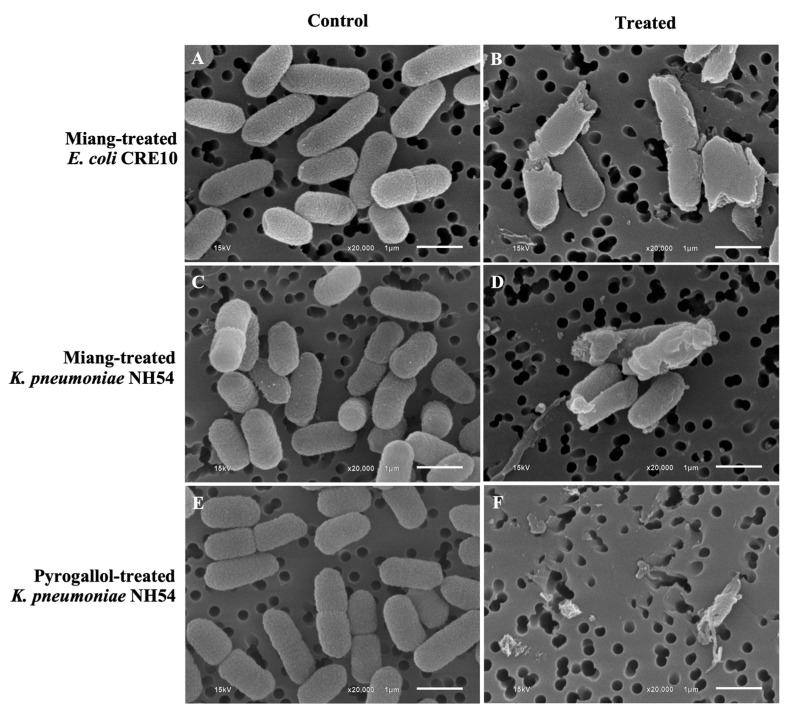
SEM images of untreated *E. coli* CRE10 and *K. pneumoniae* NH54 (**A**,**C**,**E**), after treated with *Miang* extract (**B**,**D**) and *K. pneumoniae* NH54 after treated with pyrogallol (**F**). White bars indicate 1 µm.

The *E. coli* CRE10 and *K. pneumoniae* NH54 morphological changes after exposure to *Miang* extract were examined by using scanning electron microscopy (SEM). The cell morphology changes after untreated and treated with *Miang* extract at 1 mg/mL (sub-MIC) are shown in Figure 1. After being exposed to *Miang* extract at sub-MIC for 6 h, the number of *E. coli* CRE10 and *K. pneumoniae* NH54 cells was significantly reduced compared with the untreated condition (Figure 1A,C). Both bacterial strains showed that a decrease in cell wall integrities and cell lysis were observed in both tested bacterial strains. The major morphological changes appeared to be the breakage of *E. coli* CRE10 cells (Figure 1B), while the cell wall damage and cell distortion of *K. pneumoniae* NH54 (Figure 1D) were observed. The effect of pyrogallol on *K. pneumoniae* NH54’s cell structure was found with a concentration of 0.125 mg/mL (sub-MIC). In contrast to the cells in the untreated condition (Figure 1E), the exposure of pyrogallol caused extreme damage to bacterial cells. No intact cells were observed, and only cell debris was seen (Figure 1F).

### 2.5. The Proteomic Changes in K. pneumoniae NH54 Responding to Miang Treatment

**Figure 2:**
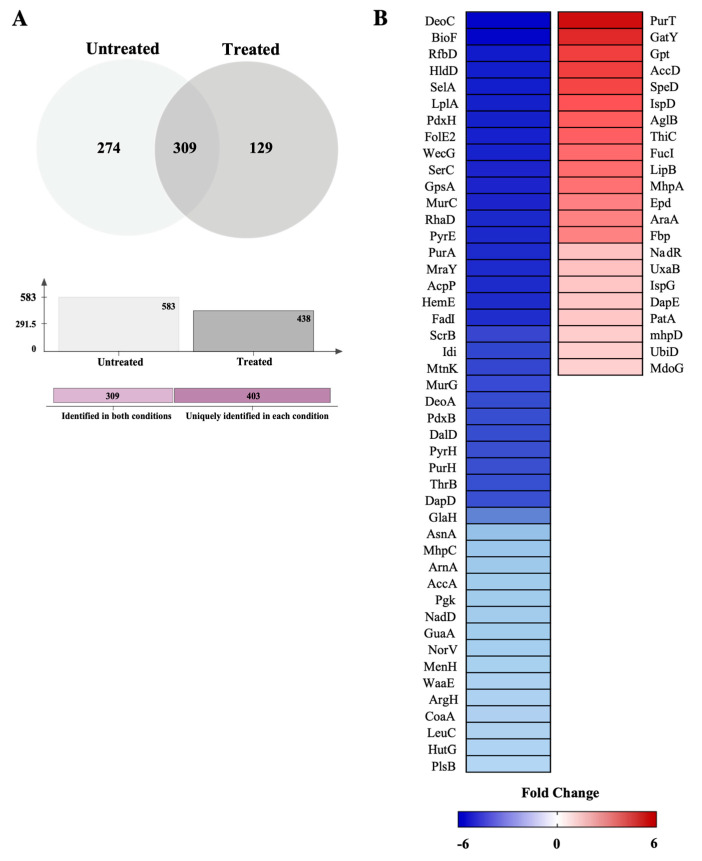
Proteomic analysis of *K. pneumoniae* NH54 responding to treatment with *Miang* extract. (**A**) Venn diagram showing number of proteins identified from untreated and treated conditions. (**B**) Heatmaps of unique significantly regulated proteins.

The effect of *Miang* extract treatment on protein expression in *K. pneumoniae* NH54 was investigated using label-free quantitative shotgun proteomics analysis. After incubation for 18 h. with 1 mg/mL (sub-MIC) of *Miang*, a total of 712 proteins was detected. A total of 274 proteins was uniquely identified in the untreated control sample, while 129 proteins were found only in the *Miang* treatment sample, and 309 proteins were expressed in both conditions, as presented in the Venn diagram (Figure 2A). The characterization of the fold change in the protein expression of *K. pneumoniae* NH54 responding to *Miang* exposure revealed that 150 proteins had a downregulated expression ranging from a 2.038- to 5.8262-fold change. The expression of 39 proteins was upregulated ranging from a 2.251- to 5.8322-fold change. The level of protein expression with known mechanisms is depicted by a heatmap (Figure 2B).

Proteins with more than a 2-fold change in expression were categorized based on their functions in biological metabolic pathways: peptidoglycan synthesis, outer membrane metabolism, carbohydrate metabolism, and amino acid metabolism (Table 4).

#### 2.5.1. The Effect on Peptidoglycan Synthesis

The initial steps of the peptidoglycan synthesis pathway taking place in the cytoplasm involve the addition of the peptide chain to N-acetylmuramate. The key step requires the activity of Uridine diphosphate (UDP)-N-acetylmuramate-L-alanine ligase or MurC for L-alanine addition to UDP-N-acetyl-alpha-D-muramate forming a peptidoglycan precursor, UDP-N-acetyl-alpha-D-muramoyl-L-alanine [6]. The series of amino acids are added to complete the linkage of a pentapeptide stem to UDP-MurNAc. The MurNAc-pentapeptide is transferred onto the lipid carrier undecaprenyl phosphate at the bacterial cytoplasmic membrane resulting in undecaprenyl-pyrophosphoryl-MurNAc-pentapeptide or lipid I. This step is catalyzed by Phospho-N-acetylmuramoyl-pentapeptide-transferase or MraY. Then, UDP-GluNAc is incorporated to lipid I by the transferase called MurG yielding undecaprenyl-pyrophosphoryl-MurNAc-pentapeptide-GlcNAc or lipid II. *K. pneumoniae* NH54 responded to *Miang* treatment by the downregulation of major enzymes, MurC, MraY, and MurG, necessary for peptidoglycan synthesis (Table 4). Based on this finding, the synthesis of peptidoglycan in *K. pneumoniae* NH54 was interrupted leading to the defect of the bacterial cell wall.

#### 2.5.2. The Effect on Outer Membrane Metabolism

The bacterial outer membrane contains lipid A and O-polysaccharide (O-antigen) as a major component of lipopolysaccharides (LPSs) which play a role as virulence factors in human pathogenesis. The consequences of *Miang* treatment led to the downregulations of *rfbD*, *wecG*, and *arnA* gene expressions (Table 4). The *rfbD* gene encodes UDP-galactopyranose mutase and catalyzes the interconversion of UDP-galactopyranose (UDP-GalP) into UDP-galactofuranose (UDP-GalF) which serves as a precursor in the biosynthesis of the galactose-containing O-side-chain polysaccharide of LPSs [7]. UDP-N-acetyl-D-mannosaminuronic acid transferase encoded by the *wecG* gene catalyzes the biosynthesis of the second lipid-linked intermediate called Und-PP-GlcNAc-ManNAcA (Lipid II) in enterobacterial common antigen (ECA) formation. ECA acts as a carbohydrate antigen found in various pathogenic strains of *Enterobacteriaceae* including *K. pneumoniae* [8]. The protein ArnA is involved in colistin (polymyxin E) resistance in various Gram-negative bacteria including *K. pneumoniae* by catalyzing the modification of lipid A with 4-amino-4-deoxy-L-arabinose (Ara4N) [9,10]. The downregulation of ArnA could reduce Ara4N addition to lipid A and retain the negative charge of the LPS. Therefore, the colistin-resistant phenotype of *K. pneumoniae* NH54 via the modification of the LPS by Ara4N addition was affected and led to the disruption of the colistin-resistant characteristic.

#### 2.5.3. The Effect on Carbohydrate Metabolism

*Miang* treatment affected various carbohydrate metabolic pathways of *K. pneumoniae* NH54. The consequences led to the upregulations of *aglB*, *araA,* and *fbp* as well as the downregulations of *scrB*, *rhaD,* and *deoC* expressions. 6-Phospho-alpha-glucosidase encoded by the *aglB* gene catalyzes the hydrolysis of maltose-6P to Glu and Glu-6P. L-arabinose isomerase (AraA) is involved in the utilization of L-arabinose as a carbon source by catalyzing the conversion of L-arabinose to L-ribulose. Fructose-1,6-bisphosphatase (Fbp), a key enzyme in the gluconeogenesis pathway, catalyzes the rate-limiting step of fructose-1,6-bisphosphate hydrolysis to fructose-6-phosphate. Hence, the upregulation of *aglB*, *araA*, and *fbp* genes suggested that *K. pneumoniae* NH54 responded to *Miang* by increasing the alternative pathways of carbon utilization. Another set of genes in which their expression levels were decreased consisted of *scrB*, *rhaD*, and *deoC* genes. Sucrose-6-phosphate hydrolase (ScrB), necessary for sucrose utilization, catalyzes the hydrolysis of the sucrose-6-phosphate glycosidic bond into glucose-6-phosphate and fructose [11]. Rhamnulose-1-phosphate aldolase (RhaD) catalyzes the reversible cleavage of L-rhamnulose-1-phosphate to L-lactaldehyde and dihydroxyacetone phosphate (DHAP) which is further used in the gluconeogenesis/glycolysis pathway. Deoxyribose-phosphate aldolase (DeoC) catalyzes the reversible reaction of the cleavage 2-deoxy-D-ribose-5-phosphate to form acetaldehyde and D-glyceraldehyde 3-phosphate which can enter the glycolysis pathway [12]. Based on this finding, *K. pneumoniae* NH54 responded to *Miang* exposure by decreasing the expressions of ScrB, RhaD, and DeoC that can lead to the lowering of glycolysis. Therefore, the common pathways of glycolysis in *K. pneumoniae* NH54 were affected.

#### 2.5.4. The Effect on Amino Acid Metabolism

The treatment of *Miang* extract leads to changes in lysine, threonine, methionine, and serine biosynthesis in *K. pneumoniae* NH54. 2,3,4,5-tetrahydropyridine-2,6-dicarboxylate N-succinyltransferase encoded by the *dapD* gene catalyzes lysine synthesis from aspartate via the diaminopimelate (DAP) pathway. However, meso-diaminopimelate, the key intermediate from this pathway, can be used for peptidoglycan synthesis [13]. Homoserine kinase (ThrB) also requires aspartate as a substrate for catalyzing threonine synthesis [14]. Methylthioribose kinase (MntK) involved in methionine biosynthesis via the salvage pathway catalyzes the phosphorylation of methylthioribose into methylthioribose-1-phosphate [15]. Phosphoserine aminotransferase (SerC) is required in the reaction of 3-phospho-serine synthesis from 3-phosphohydroxypyruvate and glutamate [16]. The proteomic data from this study showed that the expression levels of *serC*, *dapD*, *thrB*, and *mtnK* were approximately 5-fold decreased. These findings implied that *Miang* treatment considerably disturbed at least four amino acids: lysine, threonine, methionine, and serine. The decrease in the concentration of these amino acids may lead to the disturbance of the protein level in bacterial cells.

## 3. Discussion

The analysis of phenolic and flavonoid compounds in three types of fresh, streamed, and fermented Assam tea extracts revealed that pyrogallol was detectable in fermented Assam tea leaves or *Miang* extract but unidentified in fresh and steamed Assam tea leaf extracts. This finding corresponded to the report from Abdullahi et al., who suggested that pyrogallol was the product from the galloylation of phenolic biotransformation during Assam tea leaf fermentation by microorganisms [5]. Additionally, Shakya et al. demonstrated that pyrogallol was a product of *Paeoniae Radix* fermentation, an herb with anti-inflammatory activity [17]. Therefore, pyrogallol has been shown to be produced as a fermented product not only in *Miang* but also in other medicinal plants. The amounts of gallic acid also increased after the fermentation process. Gallic acid could have been produced by the activities of specific microbial enzymes for the hydrolysis of monomeric or polymeric galloylated phenolics such as flavan-3-ol polymers of other catechins, as mentioned in Abdullahi et al., 2021; Kongpichitchoke et al., 2016; and Yildiz et al., 2021 [5,18,19]. Based on this information, the production of pyrogallol as well as the increased amount of gallic acid were most likely associated with the action of microbial enzymes during *Miang* fermentation.

The antibacterial activities of the compounds in *Miang* extract were significantly higher than those in fresh and steamed Assam tea leaves due to the low MIC and MBC values. When the phenolic and flavonoid standards were tested for antibacterial activities, pyrogallol showed the highest activities against all tested bacterial strains including carbapenem- and colistin-resistant strains. Interestingly, *Miang* extract exhibited the highest antibacterial activity, while pyrogallol was identified only in this extract. This could suggest that pyrogallol was the candidate compound that played an important role in bacterial growth inhibition and the killing effect.

Pyrogallol or 1,2,3-trihydroxybenzene is commonly identified in medicinal plant extracts and has been proven to possess numerous biological activities including antioxidant, anti-inflammatory, and antibacterial activities [17,20,21,22]. The study of Taguri et al. demonstrated that polyphenols containing a pyrogallol group exhibited high antibacterial activity against various bacterial species including *S. aureus*, *E. coli*, and *K. pneumoniae* [23]. Additionally, Lim et al. exhibited that pyrogallol had activities of growth inhibition and cytotoxicity against *Vibrio vulnificus*, a human pathogen causing fatal septicemia and necrotic wound infection, by the mechanism related to polyphenol-induced pro-oxidant damage [20].

The effects of *Miang* extracts on *E. coli* CRE10 and *K. pneumoniae* NH54 cell morphologies were determined by SEM. The compounds in *Miang* extract at the MIC have the efficiency to reduce the integrity of the bacterial cell wall. Obvious results of cell structure damage occurred in both *Miang*-treated bacterial strains when compared to the untreated cells. For *K. pneumoniae* NH54, the treatment with standard pyrogallol was performed and caused a higher degree of cell damage than the results from the *Miang* treatment. After pyrogallol exposure, almost all *K. pneumoniae* NH54 cells were greatly damaged, and only cell debris was seen. The effect of pyrogallol on *S. aureus*, a Gram-positive bacterium, was observed by Chew et al. [24]. Pyrogallol caused an abnormal *S. aureus* cell shape with wrinkled surfaces, protrusion, and cell membrane disruption and reduced the *S. aureus* cell number [24]. The level of cell morphological changes responded to seemed to be different. This could be related to the higher integrity of the Gram-positive bacterial cell wall and the lower concentration of pyrogallol (31.2 μg/mL) used compared to the case of *K. pneumoniae* NH54. However, these data revealed that pyrogallol substantially disturbed bacterial cell integrity for both Gram-positive and Gram-negative bacteria.

*K. pneumoniae* NH54, extensively drug-resistant with a high colistin MIC, was selected as representative to investigate the effect of *Miang* on the protein expression level. The data from quantitative proteomic analysis displayed that the majority of identified proteins with a >2-fold change in expression were downregulated. The biological functions of these proteins were analyzed and categorized. We found that the exposure to 1 mg/mL (sub-MIC) *Miang* caused a significant effect to the expression of key enzymes involved in peptidoglycan synthesis and the metabolism of the outer membrane, carbohydrates, and amino acids in *K. pneumoniae* NH54. The consequences of *Miang* treatment may lead to the decrease in the synthesis of cell wall peptidoglycans as well as outer membrane LPSs and protein antigens resulting in the loss of bacterial cell wall integrity. This suggestion was correlated to the data from the SEM analysis in which the disruption of *K. pneumoniae* NH54 cells was seen.

The effect of *Miang* treatment on carbohydrate metabolism was complicated. However, the key enzymes, ScrB, RhaD, and DeoC, necessary for the glycolysis pathway were downregulated, while the expression of enzymes AglB, AraA, and Fbp involved in gluconeogenesis were increased. These results indicated that the exposure to Miang at sub-MIC disturbed the normal carbohydrate metabolism of *K. pneumoniae* NH54. Therefore, *K. pneumoniae* NH54 responded to the active compounds in *Miang* by using the alternative pathways for glucose synthesis to retain the energy for bacterial cell survival. The expression of four key enzymes involved in the lysine, arginine, methionine, and serine amino acid biosynthesis pathway was decreased by a >5-fold change. These results implied that the exposure to the active compound in *Miang* extract could decrease specific amino acid concentration and may lead to the disturbance of the protein level in bacterial cells.

The antibacterial activities of *C. sinensis* extracts involved in the roles of polyphenol compounds have been discovered in many studies mainly based on the determination of MIC and MBC values [25]. However, the proteomic analysis of *E. coli* ATCC 25922 conducted by Cho et al. (2007) revealed that polyphenols from Korean green tea extract caused the upregulation of proteins (GyrA, RpoS, SodC, and EmrK) involved in cellular defense and the downregulation of proteins involved in carbon and energy metabolism (Eno, SdhA, and UgpQ) and amino acid biosynthesis (GltK and TyrB) [26]. Despite the different bacterial species, the data from this study agreed with the results from our study in which *Miang* extract containing phenolic and flavonoid compounds affected the expression of enzymes in *K. pneumoniae* NH54 involved in glucose metabolism in carbohydrate biosynthesis pathways as well as amino acid biosynthesis, as discussed above.

One of the colistin resistance mechanisms is related to the modification of LPSs by 4-amino-4-deoxy-L-arabinose (Ara4N) addition. The negative charges of lipid A subunits in the LPS component of the bacterial outer membrane are commonly stabilized by binding with divalent cations, Ca^2+^ and Mg^2+^. In the presence of positively charged colistin, the interactions of divalent cations and LPSs are interfered with following the insertion of colistin molecules into the bacterial outer membrane leading to the bacterial cell wall’s disruption and finally to cell lysis. Colistin-resistant bacterial strains have developed various strategies for LPS modifications to decrease the net negativity of LPSs and reduce colistin interaction with bacterial cells. In colistin-resistant *K. pneumoniae*, the synthesis and addition of L-Ara4N to lipid A are catalyzed by the enzymes encoded in the *arnBCADTEF* operon. In colistin-susceptible strains, the expressions of the genes in this operon are repressed by negative regulators. However, the mutations of the transcriptional regulators such as MgrB, PmrD, and PmrA identified in colistin-resistant strains lead to the activation of the *arnBCADTEF* operon resulting in the addition of L-Ara4N to lipid A [27]. Interestingly, *K. pneumoniae* NH54 responded to sub-MIC *Miang* extract for 18 h. by the downregulation of AraA. The N-terminal formyltransferase domain of ArnA catalyzes the addition of a formyl group to UDP-L-Ara4N to form UDP-L-Ara4FN which is further used as a substrate in the pathway of the L-Ara4N addition of lipid A [28]. Therefore, the downregulation of AraA affects the amount of UDP-L-Ara4FN used for LPS modification. This finding implied that the compounds in *Miang* extract could disturb the mechanism of colistin resistance by reducing the positively charged modification to LPSs and lead to enhancing the colistin binding. This consequence may lead to the decrease in the colistin resistance of *K. pneumoniae* NH54.

## 4. Materials and Methods

### 4.1. Chemicals and Reagents

The HPLC-grade phenolic and flavonoid compounds were purchased from Sigma-Aldrich (St Louis, MO, USA) including (+)-catechin, (−)-gallocatechin, (−)-epigallocatechin, (−)-epicatechin, (−)-epigallocatechin gallate, (−)-gallocatechin gallate, (−)-epicatechin gallate, gallic acid, pyrogallol, ellagic acid, and caffeine.Ethanol, methanol, ethyl acetate, and ortho-phosphoric acid were supplied by Merck (Darmstadt, Germany). Analytical-grade acetic acid (Sigma-Aldrich) and HPLC-grade acetonitrile (BDH, Poole, UK) were also purchased.

### 4.2. Bacterial Strains

Antibiotic-resistant strains: methicillin-resistant *S. aureus E. coli* CRE10 and *K. pneumoniae* NH54, as well as methicillin-susceptible *S. aureus*, were collected and isolated from clinical specimens following the routine laboratory protocols by Diagnostic Laboratories of Maharaj Nakorn Chiang Mai hospital, Chiang Mai, and Nan hospital, Nan, Thailand in 2014–2021. Bacterial species were confirmed by 16S rRNA gene sequence analysis. Briefly, bacterial genomic DNA were extracted and used as DNA templates for PCR. All tested bacteria using the universal primers for 16S rRNA gene amplifications. The oligonucleotide sequences of universal primer were 27F, 5′-AGAGTTTGATCMTGGCTCAG-3′ and 1492r, 5′-CGGTTACCTTGTTACGACTT-3′ [29]. PCR was performed using a thermal cycler (Biometra, Gottingen, Germany) with the following protocol: initial denaturation at 95 °C for 10 min; 30 cycles of denaturation at 95 °C for 30 s; annealing at 52 °C for 40 s; extension at 72 °C for 1 min; and final extension at 72 °C for 5 min. PCR amplicons with approximately 1400 bp were sent to a sequencing service (Macrogen, Seoul, Republic of Korea). The obtained nucleotide sequences were searched for sequence similarity using the Basic Local Alignment Search Tool (https://blast.ncbi.nlm.nih.gov/Blast.cgi, accessed on 10 June 2023). The results confirmed bacterial species as *S. aureus*, *E. coli*, and *K. pneumoniae*. Antibiotic-susceptible control strain: *E. coli* ATCC 25922 was purchased (Thermo Scientific, Winsford, UK).

### 4.3. Sample Extraction

Fresh and streamed Assam tea leaves (steamed for 2 h and cooled down at room temperature) were collected from Pang Ma-O village, Chiang Dao district, Chiang Mai, Thailand (Latitude 19.27750, Longitude 98.90367), in November 2019. For *Miang*, the non-filamentous fungi-based fermentation process was conducted as described by Khanongnuch et al. (2021) [3]. All samples were dried at 50 °C for 24 h and extracted by The80% ethanol at 60 °C in a shaking incubator for 1 h according to Wangkarn et al. (2021) [30]. The extracted solution was filtered through filter paper No. 1 and evaporated under reduced pressure and dried with a vacuum dryer.

### 4.4. Chromatographic Analysis of Catechin, Catechin Derivatives, and Related Compounds

The quantity of catechin, its derivatives, and related compounds in fresh, streamed, and fermented Assam tea leaves (*Miang*) was analyzed by HPLC according to the condition of Wangkarn et al. (2021) [30]. Briefly, the liquid chromatography system (HP 1200 series, Agilent Technologies, Santa Clara, CA, USA) coupled with a multiwavelength detector was used. A reversed-phase LC column namely Symmetry RP-18 column (4.6 × 250 mm, 5 µm particle size, Waters, MA, USA) equipped with a specific C18 guard column was used with a mobile phase consisting of 0.1% acetic acid in acetronitrile and 0.1% acetic acid in DI water at a flow rate of 1.0 mL/min. The detection of analytes was performed by UV detection at 210 and 278 nm. All samples were analyzed in triplicate.

### 4.5. Antibiotic Susceptibility Testing

The susceptibility to twenty-seven antibiotic agents classified into sixteen classes is listed in Table S1. The determination was investigated by the disc diffusion method on Muller–Hinton agar except for colistin susceptibility which was carried out by the broth microdilution test. Twenty antibiotic agents were tested particularly against Gram-negative bacteria, whereas nine antibiotic agents were specifically for testing against Gram-positive bacteria. *S. aureus* MSSA01 and *E. coli* ATCC 25922 susceptible to all tested antibiotic agents were used as Gram-positive and Gram-negative reference strains, respectively. The results were interpreted according to CLSI clinical breakpoint guidelines 2021 [31].

### 4.6. Minimum Inhibitory Concentration (MIC) and Minimum Bactericidal Concentration (MBC) of Tested Extracts and Phenolic and Flavonoid Compounds

Dried extracts of fresh, streamed, and fermented Assam tea leaves prepared as mentioned above were resuspended in 5% DMSO. The solution of phenolic and flavonoid compounds, including (+)-catechin, (−)-gallocatechin, (−)-epigallocatechin, (−)-epicatechin, (−)-epigallocatechin gallate, (−)-gallocatechin gallate, (−)-epicatechin gallate, gallic acid, pyrogallol, ellagic acid, and caffeine, was prepared by dissolving in 5% DMSO. The MICs of tested extracts and phenolic and flavonoid compounds against multidrug-resistant bacteria were determined by a broth microdilution assay. A total of 50 μL of either tested extract or phenolic and flavonoid compounds was mixed with 50 μL Muller–Hinton broth in a microtiter plate and serially 2-fold diluted ranging from 128 to 0.5 mg/mL for tested extracts and from 2 to 0.125 mg/mL for phenolic and flavonoid compounds. Bacterial growth was determined by the examination of visual turbidity after incubation at 37 °C for 18 h. The MIC of either the tested extract or phenolic and flavonoid compounds was recorded. The mixture from the well with no bacterial growth was taken and spread on Muller–Hinton agar for the determination of the MBC. After the incubation at 37 °C for 18 h, the concentration of the tested extract or phenolic and flavonoid compounds that caused the absence of bacterial growth was recorded as the MBC.

### 4.7. Determination of Bacterial Cell Damage by Scanning Electron Microscopy (SEM)

The effect of *Miang* extract on bacterial cell morphology was examined by using scanning electron microscopy (SEM). Colistin-resistant bacteria: *E. coli* CRE10 and *K. pneumoniae* NH54 were subjected for *Miang* extract treatment, and only *K. pneumoniae* NH54 was tested with pyrogallol. Briefly, a single bacterial colony of each strain was sub-cultured in Luria–Bertani (LB) broth (Difco, Sparks, MD, USA) and incubated at 37 °C with shaking at 150 rpm until the growth reached an OD_600_ of 0.4. Bacterial culture was treated with *Miang* extract or pyrogallol at the concentration of the sub-MIC and further incubated at 37 °C. After 6 h. of incubation, bacterial cells were harvested by centrifugation at 4000 rpm, 10 min at 4 °C. Cell pellets were washed three times with 0.1 M phosphate buffer pH 7.4 and filtered with Isopore™ Polycarbonate Membrane Filters, 0.2 μm. The bacterial cells on the filters were dehydrated in ethanol series (50%, 75%, 85%, 95%, and 100%) by adding each ethanol solution twice for 15 min. The samples were dried using a Quorum K850 critical point dryer (Quorum Technologies, Lewes, UK). The samples were mounted on standard aluminum stubs followed by coating with gold using a Q150R plus-rotary pumped coater (Quorum Technologies, Lewes, UK). Each sample was prepared for SEM analysis in duplicate. Micrographs were taken by a JSM-6610LV scanning electron microscope (JEOL, Tokyo, Japan).

### 4.8. Sample Preparation for Shotgun Proteomics

Bacterial cultures of *K. pneunoniae* NH54 without and with 1 mg/mL (sub-MIC) Miang extract were incubated at 37 °C. After 18 h. incubation, the total protein was prepared from 1 OD_600_ (2 mL) cells by centrifugation at 12,000 rpm for 5 min, washed twice with 1 mL doubled-distilled water, the pellet was resuspended in 100 μL of 0.5% SDS, and the protein content was measured with Lowry assay using bovine serum albumin as a protein standard [32]. Five micrograms of each bacterial protein sample was subjected to in-solution digestion. Samples were completely dissolved in 10 mM ammonium bicarbonate, reduced with 5 mM dithiothreitol (DTT) at 60 °C for 1 h, and alkylated with 15 mM iodoacetamide (IAA) at room temperature for 45 min in the dark. Trypsin (mass spectrometry grade, Promega, Madison, WI, USA) was added in a 1:20 ratio and incubated at 37 °C for 16 h. Prior to LC-MS/MS analysis, the digested samples were dried and redissolved with 0.1% formic acid followed by injection into LC-MS/MS.

### 4.9. Liquid Chromatography–Tandem Mass Spectrometry (LC/MS-MS)

The tryptic peptide samples were injected into LC-MS for analysis. One microliter of the peptide samples was injected into an Acclaim PepMap RSLC C18 column (75 μm I.D. × 15 cm, 2 μm particle size, 100 Å pore size (Thermo Scientific, Winsford, UK) of an Ultimate3000 Nano/Capillary LC System (Thermo Scientific, Winsford, UK) equipped with a Hybrid quadrupole Q-Tof impact II™ (Bruker Daltonics, Billerica, MA, USA). Solvent A was composed of 0.1% (*v*/*v*) formic acid (FA) in water, whereas solvent B was a solution of 0.1% (*v*/*v*) FA in 80% (*v*/*v*) acetonitrile. A gradient of 5–55% solvent B was used to elute the peptides at a constant flow rate of 0.30 μL/min for 30 min. The column temperature was maintained at 60 °C. Nitrogen was used as a drying gas at a flow rate of 50 L/h. Analysis was performed in positive polarity mode with a spray voltage of 1.6 kV. The mass-to-charge ratio (*m*/*z*) was set between 150 and 2200 Da. Collision-induced dissociation (CID) product ion mass spectra were generated using nitrogen as the collision gas. The collision energy was adjusted to 10 eV in response to the *m*/*z* value. The LC-MS analysis of each sample was performed in triplicate.

### 4.10. Bioinformatics and Data Analysis

The data files acquired from LC-Q-Tof MS were processed using MaxQuant 2.2.0.0 [33]. Proteins were identified through searches against Uniprot *E. coli* and *Campylobacter* spp. databases. The significance threshold for protein identification was established with a *p*-value < 0.05 and a false discovery rate (FDR) of 1%. The specific parameters for MaxQuant’s standard configuration encompassed allowing for a maximum of two missed cleavages, setting the main search mass tolerance at 0.6 daltons, utilizing trypsin as the enzyme for digestion, applying a fixed modification of cysteine through carbamidomethylation, and incorporating variable modifications for methionine oxidation and protein N-terminus acetylation. Peptides were considered for identification and subsequent data analysis if they met the criteria of being at least seven amino acids in length and containing at least one unique peptide. Ion intensities were log2-transformed, and missing values were imputed by Perseus 1.6.6.0 [34] using a constant value (zero). The visualization of the LC-MS data (heatmap) was conducted using Metaboanalyst [35]. The functions of proteins were investigated by Panther [36]. A Venn diagram was used to depict identified proteins found in untreated and treated conditions [37].

## 5. Conclusions

In this study, the effects on the cell morphology and the changes in the protein expression of XDR *K. pneumoniae* NH54 responding to *Miang* were examined and led to the better understanding of its antibacterial mechanism. Pyrogallol was detected only in fermented Assam tea leaves (*Miang*) and showed efficiency as an antibacterial compound against both antibiotic-susceptible and -resistant strains. The compounds in *Miang* extract containing pyrogallol at the MIC have the efficiency to reduce the integrity of the bacterial cell wall as demonstrated in the data from the SEM analysis. One of the most interesting data was revealed at sub-MIC *Miang* exposure: the downregulation of AraA which is the enzyme involved in LPS modification and related to the colistin resistance mechanism in Gram-negative bacteria. This finding implied that the exposure to compounds in *Miang* extract could disturb the mechanism of colistin resistance mediated by the decrease in the net negativity of LPSs leading to reducing colistin interaction with bacterial cells. Based on the outcome of this research, we suggest that the presence of pyrogallol in *Miang* extract as a secondary metabolite in combination with other phenolic and flavonoid compounds from fermentation could enhance the antibacterial activity of *Miang*.

## Figures and Tables

**Table 1:** Amount of catechin, catechin derivatives, and related compounds in fresh, streamed, and fermented Assam tea leaf (*Miang*) extracts.

Compounds	Bioactive Compounds (mg/g Extract)		
	Fresh Assam Tea Leaves	Streamed Assam Tea Leaves	Fermented Assam Tea Leaves
Gallic acid	0.94 ± 0.05	1.42 ± 0.07	2.45 ± 0.08
Pyrogallol	ND	ND	4.37 ± 0.06
Gallocatechin	3.65 ± 0.09	3.47 ± 0.09	2.73 ± 0.12
Epigallocatechin	8.84 ± 0.13	3.76 ± 0.13	4.52 ± 0.15
Catechin	36.78 ± 0.18	22.26 ± 0.17	17.75 ± 0.13
Caffeine	17.37 ± 0.11	23.62 ± 0.12	15.45 ± 0.09
Epicatechin	5.32 ± 0.12	3.18 ± 0.07	6.12 ± 0.08
Epigallocatechin gallate	3.71 ± 0.10	3.85 ± 0.08	2.96 ± 0.11
Gallocatechin gallate	1.18 ± 0.07	0.92 ± 0.05	1.43 ± 0.08
Epicatechin gallate	1.83 ± 0.09	2.26 ± 0.10	1.37 ± 0.08

Mean ± SD; ND = not detected (below limit of detection value).

**Table 2:** Minimum inhibitory concentration and minimum bactericidal concentration of tested extracts against antibiotic-resistant bacteria.

Bacterial Isolates	Fresh Assam Tea Leaves (mg/mL)		Steamed Assam Tea Leaves (mg/mL)		Fermented Assam Tea Leaves (mg/mL)	
	MIC	MBC	MIC	MBC	MIC	MBC
*E. coli* CRE10	32	64	8	8	2	2
*K. pneumoniae* NH54	32	64	8	8	2	2
*S. aureus* MRSA08	8	8	1	1	0.5	1
*S. aureus* MSSA01	8	8	1	1	1	2
*E. coli* ATCC 25922	32	64	16	16	2	2

**Table 3:** Minimum inhibitory concentration and minimum bactericidal concentration of phenolic and flavonoid compounds against antibiotic-resistant bacteria.

Bacterial Isolates	Epicatechin (mg/mL)		Catechin (mg/mL)		Epigallocatechin Gallate (mg/mL)		Pyrogallol (mg/mL)		Gallic Acid (mg/mL)		Ellagic Acid (mg/mL)		Caffeine (mg/mL)	
	MIC	MBC	MIC	MBC	MIC	MBC	MIC	MBC	MIC	MIC	MIC	MBC	MIC	MBC
*E. coli* CRE10	>2	>2	>2	>2	>2	>2	0.25	0.5	>2	>2	>2	>2	>2	>2
*K. pneumoniae* NH54	>2	>2	>2	>2	>2	>2	0.25	0.5	>2	>2	>2	>2	>2	>2
*S. aureus* MRSA08	>2	>2	>2	>2	0.5	2	0.25	0.5	>2	>2	>2	>2	>2	>2
*S. aureus* MSSA01	>2	>2	>2	>2	0.5	1	0.25	0.5	>2	>2	>2	>2	>2	>2
*E. coli* ATCC 25922	>2	>2	>2	>2	>2	>2	0.25	0.25	>2	>2	>2	>2	>2	>2

**Table 4:** Proteins with expression level changes responding to *Miang* extract treatment.

Protein ID	Gene	Description	Pathway	Expression Level (Fold Change)
Peptidoglycan biogenesis				
B5Y1U7	*murG*	UDP-N-acetylglucosamine-N-acetylmuramyl-(pentapeptide) pyrophosphoryl undecaprenol N-acetylglucosamine transferase	Peptidoglycan biosynthesis.	Down (5.0832)
A6T4N0	*mraY*	Phospho-N-acetylmuramoyl-pentapeptide-transferase	Peptidoglycan biosynthesis.	Down (5.4706)
A6T4N4	*murC*	UDP-N-acetylmuramate-L-alanine ligase	Peptidoglycan biosynthesis.	Down (5.5112)
Outer membrane metabolism				
A6TF98	*arnA*	Bifunctional polymyxin resistance protein ArnA	LPS modification by the modification of lipid A with 4-amino-4-deoxy-L-arabinose (Ara4N) required for resistance to polymyxin and cationic antimicrobial peptides.	Down (2.6563)
B5XYX5	*wecG*	UDP-N-acetyl-D-mannosaminuronic acid transferase	The biosynthesis of Und-PP-GlcNAc-ManNAcA (Lipid II), the second lipid-linked intermediate involved in enterobacterial common antigen (ECA) synthesis.	Down (5.5389)
Q48485	*rfbD*	UDP-galactopyranose mutase	LPS O-antigen biosynthesis. Involved in the biosynthesis of the galactose-containing O-side-chain polysaccharide backbone structure of D-galactan I, a key component of LPSs.	Down (5.6201)
Carbohydrate metabolism				
Q9AGA6	*aglB*	6-phospho-alpha-glucosidase	Catalyzes the hydrolysis of maltose-6P to Glu and Glu-6P.	Up (5.3427)
B5Y1Y1	*araA*	L-arabinose isomerase	Catalyzes the conversion of L-arabinose to L-ribulose.	Up (5.0525)
B5Y2X3	*fbp*	Fructose-1,6-bisphosphatase class 1	Gluconeogenesis (F 1,6 BP converted to F6P to G6P in Gluconeogenesis).	Up (5.0497)
P27217	*scrB*	Sucrose-6-phosphate hydrolase	Glycosidic bond hydrolysis.	Down (5.1606)
A6TGA6	*rhaD*	Rhamnulose-1-phosphate aldolase	L-rhamnose degradation to DHAP and L-lactaldehyde.	Down (5.4894)
B5Y277	*deoC*	Deoxyribose-phosphate aldolase	2-deoxy-D-ribose 1-phosphate formation from Gly-3P and acetaldehyde.	Down (5.8262)
Amino-acid biosynthesis				
B5Y1K5	*dapD*	2,3,4,5-tetrahydropyridine-2,6-dicarboxylate N-succinyltransferase	L-lysine biosynthesis via the DAP pathway.	Down (5.0427)
A6T4E3	*thrB*	Homoserine kinase	L-threonine biosynthesis; L-threonine from L-aspartate.	Down (5.0516)
Q9F0P1	*mtnK*	Methylthioribose kinase	L-methionine biosynthesis via the salvage pathway.	Down (5.1368)
B5XY88	*serC*	Phosphoserine aminotransferase	L-serine biosynthesis; L-serine from 3-phospho-D-glycerate.	Down (5.5224)

## Data Availability

Data are available upon request.

## Supplementary Materials

The following supporting information can be downloaded at https://www.mdpi.com/article/10.3390/antibiotics13060536/s1, Figure S1: HPLC chromatogram of mixed standards and *Miang* extract; Table S1: Antibiotic susceptibility of antibiotic-resistant bacteria and antibiotic-susceptible control strains.

## References

[1] World Health Organization (2017). Prioritization of Pathogens to Guide Discovery, Research and Development of New Antibiotics for Drug-Resistant Bacterial Infections, Including Tuberculosis. World Health Organization. https://iris.who.int/handle/10665/311820.

[2] Kawakami M., Chairote G., Kobayashi A. (1987). Flavor Constituents of Pickled Tea, Miang, in Thailand. Agric. Biol. Chem..

[3] Khanongnuch C., Unban K., Kanpiengjai A., Saenjum C. (2017). Recent research advances and ethno-botanical history of *miang*, a traditional fermented tea (*Camellia sinensis* var. *assamica*) of northern Thailand. J. Ethn. Foods.

[4] Youyi H., Cong L., Xiudan X. (2016). Quality Characteristics of a Pickled Tea Processed by Submerged Fermentation. Int. J. Food Prop..

[5] Abdullahi A.D., Kodchasee P., Unban K., Pattananandecha T., Saenjum C., Kanpiengjai A., Shetty K., Khanongnuch C. (2021). Comparison of Phenolic Contents and Scavenging Activities of Miang Extracts Derived from Filamentous and Non-Filamentous Fungi-Based Fermentation Processes. Antioxidants.

[6] Egan A.J.F., Errington J., Vollmer W. (2020). Regulation of peptidoglycan synthesis and remodelling. Nat. Rev. Microbiol..

[7] Whitfield C., Williams D.M., Kelly S.D. (2020). Lipopolysaccharide O-antigens-bacterial glycans made to measure. J. Biol. Chem..

[8] Rai A.K., Mitchell A.M. (2020). Enterobacterial Common Antigen: Synthesis and Function of an Enigmatic Molecule. mBio.

[9] Gogry F.A., Siddiqui M.T., Sultan I., Haq Q.M.R. (2021). Current Update on Intrinsic and Acquired Colistin Resistance Mechanisms in Bacteria. Front. Med..

[10] Breazeale S.D., Ribeiro A.A., McClerren A.L., Raetz C.R. (2005). A formyltransferase required for polymyxin resistance in *Escherichia coli* and the modification of lipid A with 4-Amino-4-deoxy-L-arabinose. Identification and function oF UDP-4-deoxy-4-formamido-L-arabinose. J. Biol. Chem..

[11] Titgemeyer F., Jahreis K., Ebner R., Lengeler J.W. (1996). Molecular analysis of the scrA and scrB genes from *Klebsiella pneumoniae* and plasmid pUR400, which encode the sucrose transport protein Enzyme II Scr of the phosphotransferase system and a sucrose-6-phosphate invertase. Mol. Gen. Genet. MGG.

[12] Chambre D., Guérard-Hélaine C., Darii E., Mariage A., Petit J.L., Salanoubat M., de Berardinis V., Lemaire M., Hélaine V. (2019). 2-Deoxyribose-5-phosphate aldolase, a remarkably tolerant aldolase towards nucleophile substrates. Chem. Commun..

[13] Rodionov D.A., Vitreschak A.G., Mironov A.A., Gelfand M.S. (2003). Regulation of lysine biosynthesis and transport genes in bacteria: Yet another RNA riboswitch?. Nucleic Acids Res..

[14] Huo X., Viola R.E. (1996). Substrate specificity and identification of functional groups of homoserine kinase from *Escherichia coli*. Biochemistry.

[15] North J.A., Wildenthal J.A., Erb T.J., Evans B.S., Byerly K.M., Gerlt J.A., Tabita F.R. (2020). A bifunctional salvage pathway for two distinct S-adenosylmethionine by-products that is widespread in bacteria, including pathogenic *Escherichia coli*. Mol. Microbiol..

[16] Duncan K., Coggins J.R. (1986). The serC-aro A operon of *Escherichia coli*. A mixed function operon encoding enzymes from two different amino acid biosynthetic pathways. Biochem. J..

[17] Shakya S., Danshiitsoodol N., Sugimoto S., Noda M., Sugiyama M. (2021). Anti-Oxidant and Anti-Inflammatory Substance Generated Newly in Paeoniae Radix Alba Extract Fermented with Plant-Derived *Lactobacillus brevis* 174A. Antioxidants.

[18] Kongpichitchoke T., Chiu M.T., Huang T.C., Hsu J.L. (2016). Gallic Acid Content in Taiwanese Teas at Different Degrees of Fermentation and Its Antioxidant Activity by Inhibiting PKCδ Activation: In Vitro and in Silico Studies. Molecules.

[19] Yildiz E., Guldas M., Gurbuz O. (2021). Determination of in-vitro phenolics, antioxidant capacity and bio-accessibility of Kombucha tea produced from black carrot varieties grown in Turkey. Food Sci. Technol..

[20] Lim J.Y., Kim C.M., Rhee J.H., Kim Y.R. (2016). Effects of Pyrogallol on Growth and Cytotoxicity of Wild-Type and katG Mutant Strains of *Vibrio vulnificus*. PLoS ONE.

[21] Ozturk Sarikaya S.B. (2015). Acethylcholinesterase inhibitory potential and antioxidant properties of pyrogallol. J. Enzym. Inhib. Med. Chem..

[22] Lima V.N., Oliveira-Tintino C.D., Santos E.S., Morais L.P., Tintino S.R., Freitas T.S., Geraldo Y.S., Pereira R.L., Cruz R.P., Menezes I.R. (2016). Antimicrobial and enhancement of the antibiotic activity by phenolic compounds: Gallic acid, caffeic acid and pyrogallol. Microb. Pathog..

[23] Taguri T., Tanaka T., Kouno I. (2006). Antibacterial spectrum of plant polyphenols and extracts depending upon hydroxyphenyl structure. Biol. Pharm. Bull..

[24] Chew Y.L., Arasi C., Goh J.K. (2022). Pyrogallol induces antimicrobial effect and cell membrane disruption on methicillin-resistant *Staphylococcus aureus* (MRSA). Curr. Bioact. Compd..

[25] Zhao T., Li C., Wang S., Song X. (2022). Green Tea (*Camellia sinensis*): A Review of Its Phytochemistry, Pharmacology, and Toxicology. Molecules.

[26] Cho Y.S., Schiller N.L., Kahng H.Y., Oh K.H. (2007). Cellular responses and proteomic analysis of *Escherichia coli* exposed to green tea polyphenols. Curr. Microbiol..

[27] Olaitan A.O., Morand S., Rolain J.M. (2014). Mechanisms of polymyxin resistance: Acquired and intrinsic resistance in bacteria. Front. Microbiol..

[28] Yan A., Guan Z., Raetz C.R. (2007). An undecaprenyl phosphate-aminoarabinose flippase required for polymyxin resistance in *Escherichia coli*. J. Biol. Chem..

[29] Lane D.J., Stackebrandt E., Goodfellow M. (1991). 16S/23S rRNA sequencing. Nucleic Acid Techniques in Bacterial Systematics.

[30] Wangkarn S., Grudpan K., Khanongnuch C., Pattananandecha T., Apichai S., Saenjum C. (2021). Development of HPLC Method for Catechins and Related Compounds Determination and Standardization in Miang (Traditional Lanna Fermented Tea Leaf in Northern Thailand). Molecules.

[31] CLSI (2021). Performance Standards for Antimicrobial Susceptibility Testing, M100.

[32] Lowry O.H., Rosebrough N.J., Farr A.L., Randall R.J. (1951). Protein measurement with the Folin phenol reagent. J. Biol. Chem..

[33] Tyanova S., Temu T., Cox J. (2016). The MaxQuant computational platform for mass spectrometry-based shotgun proteomics. Nat. Protoc..

[34] Tyanova S., Temu T., Sinitcyn P., Carlson A., Hein M.Y., Geiger T., Mann M., Cox J. (2016). The Perseus computational platform for comprehensive analysis of (prote)omics data. Nat. Methods.

[35] Pang Z., Zhou G., Ewald J., Chang L., Hacariz O., Basu N., Xia J. (2022). Using MetaboAnalyst 5.0 for LC-HRMS spectra processing, multi-omics integration and covariate adjustment of global metabolomics data. Nat. Protoc..

[36] Mi H., Muruganujan A., Ebert D., Huang X., Thomas P.D. (2019). PANTHER version 14: More genomes, a new PANTHER GO-slim and improvements in enrichment analysis tools. Nucleic Acids Res..

[37] Bardou P., Mariette J., Escudié F., Djemiel C., Klopp C. (2014). jvenn: An interactive Venn diagram viewer. BMC Bioinform..

