# Supplementary material for: Antibacterial Activities of Phenolic Compounds in Miang Extract: Growth Inhibition and Change in Protein Expression of Extensively Drug-Resistant Klebsiella pneumoniae
Source: Antibiotics (Basel). 2024 Jun 9;13(6):536. doi: 10.3390/antibiotics13060536 (PMC11201136; doi:10.3390/antibiotics13060536)
Supplement: Supplementary file 1 [file antibiotics-13-00536-s001.zip › antibiotics-3030145-supplementary.pdf]

Supplementary Materials

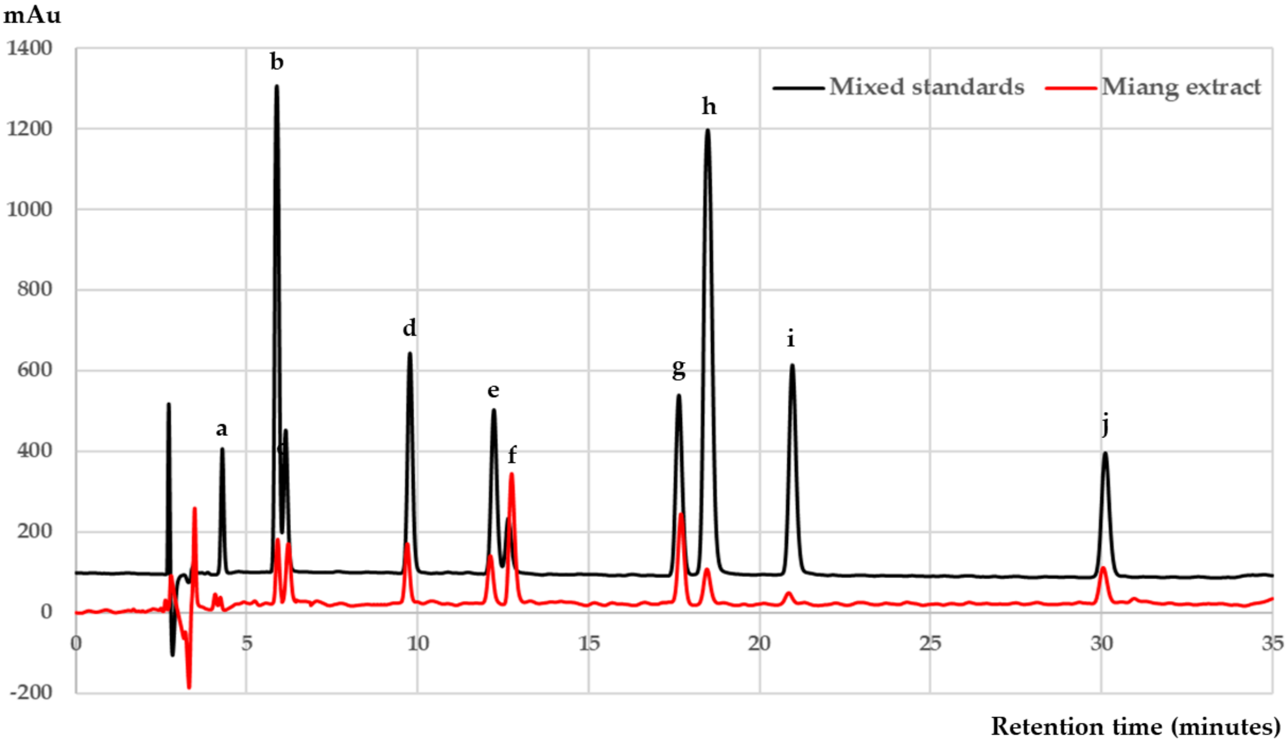

Figure S1. HPLC chromatogram of mixed standards and *Miang* extract. The peaks indicate (a) gallic acid, (b) pyrogallol, (c) gallocatechin, (d) epigallocatechin, (e) catechin, (f) caffeine, (g) epicatechin, (h) epigallocatechin gallate, (i) gallocatechin gallate, and (j) epicatechin gallate.

Table S1. Antibiotic susceptibility of antibiotic resistant bacteria and antibiotic susceptible-control strains.

**Antibiotic Class**	**Antibiotic Agent**	***E. coli* *CRE10***	***K. pneumoniae* *NH54***	***S. aureus* MRSA08**	***S. aureus* MSSA01**	***E. coli* ATCC25922**
Macrolide	Erythromycin *	—	—	R	S	—
Oxazolidinones	Linezolid *	—	—	S	S	—
Glycopeptide	Vancomycin *	—	—	S	S	—
Lincosamines	Clindamycin *	—	—	R	S	—
Penicillins	Ampicillin	R	R	—	—	S
	Oxacillin *	—	—	R	S	—
β-Lactam/ β-lactamase inhibitor	Amoxicillin/clavu- lanic acid	R	R	—	—	S
Cephems	Cefazolin	R	R	—	—	S
	Cefuroxime	R	R	—	—	S
	Cefoxitin	R	S	R	S	S
	Cefotaxime	R	R	—	—	S
	Ceftazidime	R	R	—	—	S
Carbapenems	Imipenem	R	S	—	—	S
	Meropenem	R	R	—	—	S
	Ertapenem	R	R	—	—	S
Aminoglycoside	Gentamicin	R	S	R	S	S
	Amikacin	R	S	—	—	S
Monobactam	Aztreonam	R	R	—	—	S
Fluoroquinolones	Ciprofloxacin	R	R	—	—	S
	Moxifloxacin *	—	—	R	S	—
Folate pathway inhibitors	Trimethoprim/sulfa- methoxazole	R	R	R	S	S
Phenocols	Chloramphenicol	S	S	—	—	S
Tetracyclines	Tetracycline	R	R	—	—	S
	Tigecycline	S	S	—	—	S
Fosfomycins	Fosfomycin	R	R	—	—	S
Lipopeptides	Colistin (MIC, ug/ml)	R, 4	R, 16	—	—	S, ≤ 2

* Antimicrobial agents for gram positive bacterial susceptibility testing.
